# Growth Rate and Acceleration Analysis of the COVID-19 Pandemic Reveals the Effect of Public Health Measures in Real Time

**DOI:** 10.3389/fmed.2020.00247

**Published:** 2020-05-22

**Authors:** Yuri Tani Utsunomiya, Adam Taiti Harth Utsunomiya, Rafaela Beatriz Pintor Torrecilha, Silvana de Cássia Paulan, Marco Milanesi, José Fernando Garcia

**Affiliations:** ^1^Department of Support, Production and Animal Health, School of Veterinary Medicine of Araçatuba, São Paulo State University (Unesp), Araçatuba, Brazil; ^2^International Atomic Energy Agency (IAEA) Collaborating Centre on Animal Genomics and Bioinformatics, Araçatuba, Brazil; ^3^Department of Preventive Veterinary Medicine and Animal Reproduction, School of Agricultural and Veterinarian Sciences, São Paulo State University (Unesp), Jaboticabal, Brazil

**Keywords:** coronavirus, severe acute respiratory syndrome, growth curve analysis, mathematical modeling, moving regression, Hidden Markov Model

## Abstract

**Background:** Ending the COVID-19 pandemic is arguably one of the most prominent challenges in recent human history. Following closely the growth dynamics of the disease is one of the pillars toward achieving that goal.

**Objective:** We aimed at developing a simple framework to facilitate the analysis of the growth rate (cases/day) and growth acceleration (cases/day^2^) of COVID-19 cases in real-time.

**Methods:** The framework was built using the Moving Regression (MR) technique and a Hidden Markov Model (HMM). The dynamics of the pandemic was initially modeled via combinations of four different growth stages: lagging (beginning of the outbreak), exponential (rapid growth), deceleration (growth decay), and stationary (near zero growth). A fifth growth behavior, namely linear growth (constant growth above zero), was further introduced to add more flexibility to the framework. An *R Shiny* application was developed, which can be accessed at https://theguarani.com.br/ or downloaded from https://github.com/adamtaiti/SARS-CoV-2. The framework was applied to data from the European Center for Disease Prevention and Control (ECDC), which comprised 3,722,128 cases reported worldwide as of May 8th 2020.

**Results:** We found that the impact of public health measures on the prevalence of COVID-19 could be perceived in seemingly real-time by monitoring growth acceleration curves. Restriction to human mobility produced detectable decline in growth acceleration within 1 week, deceleration within ~2 weeks and near-stationary growth within ~6 weeks. Countries exhibiting different permutations of the five growth stages indicated that the evolution of COVID-19 prevalence is more complex and dynamic than previously appreciated.

**Conclusions:** These results corroborate that mass social isolation is a highly effective measure against the dissemination of SARS-CoV-2, as previously suggested. Apart from the analysis of prevalence partitioned by country, the proposed framework is easily applicable to city, state, region and arbitrary territory data, serving as an asset to monitor the local behavior of COVID-19 cases.

## Introduction

The World Health Organization (WHO) officially declared Coronavirus Disease (COVID-19) a global pandemic on March 11th 2020 (1). The disease is caused by the novel Severe Acute Respiratory Syndrome Coronavirus 2 (SARS-CoV-2) (2, 3), which seems to have first emerged in Wuhan, China on December 12th 2019 (4, 5). Worldwide dissemination has been extremely rapid, and by the time this study was completed (May 8th 2020) a total of 3,722,128 cases and 263,288 deaths had been reported across 209 countries and territories according to data from the European Center for Disease Prevention and Control (ECDC) (6). Approximately 86% of all cases are estimated to have been undocumented prior to the cordon sanitaire in China (7), which suggests that the disease might be also substantially under-reported in other countries. Nevertheless, partial COVID-19 prevalence data are still an invaluable resource to help monitoring and controlling the disease. In particular, extracting daily estimates of growth rate (cases/day) and acceleration (cases/day^2^) in disease dissemination from real-time case reports can be decisive for an effective and promptly action to restrain further contagion. Here we report the development of a simple framework dedicated to the real-time analysis of COVID-19 prevalence. This framework was built using a combination of Moving Regression (MR) (8) and Hidden Markov Model (HMM) (9), and was deployed as a *Shiny* (10) application in *R* (11). Here we show the utility of that framework in the analysis of publicly available COVID-19 case reports that are updated daily by the ECDC. The scope of the framework was to provide real-time extractions of growth rates and acceleration from prevalence data, as well as to provide automated classification of growth stages. Accurate predictions of next-day cases were also obtained as a secondary product.

## Results and Discussion

**Figure 1:**
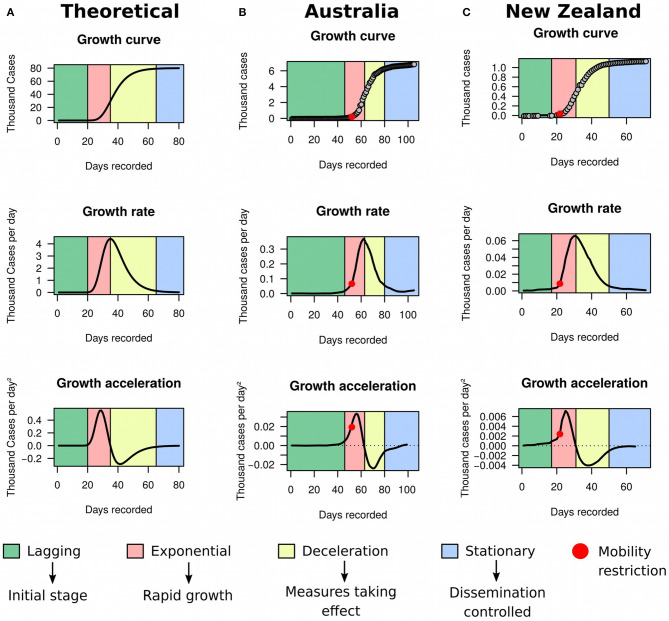
Growth rate and acceleration in Australia and New Zealand. **(A)** Theoretical model exemplified by simulated data using a three-parameters Gompertz model with an asymptote at 80,000, growth coefficient of 0.15, inflection time at 35, and time ranging from 1 to 80. **(B)** Fitted curves for Australia between January 25th and May 8th 2020. **(C)** Fitted curves for New Zealand between February 28th and May 8th 2020.

For simplicity, assume that the cumulative number of COVID-19 cases over time (i.e., the growth curve of prevalence) in a specific country or territory follows an unknown sigmoidal function (Figure 1A). Such assumption is common in the analysis of growth data and has been applied to a wide range of problems, from tumor (12) to bacterial (13) growth. Although empirical data from a number of countries—including Australia (Figure 1B) and New Zealand (Figure 1C)—seemed to support it well, that assumption will be substantially relaxed later in our framework to accommodate complex dynamics in the evolution of COVID-19 prevalence.

**Figure 2:**
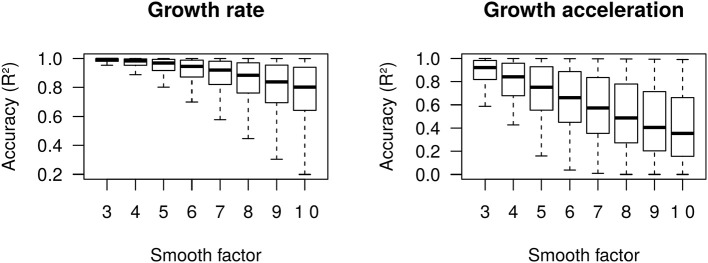
Accuracy (*R*^2^) of moving regression estimates of growth rate and growth acceleration from 50,000 simulated Gompertz growth curves.

**Figure 3:**
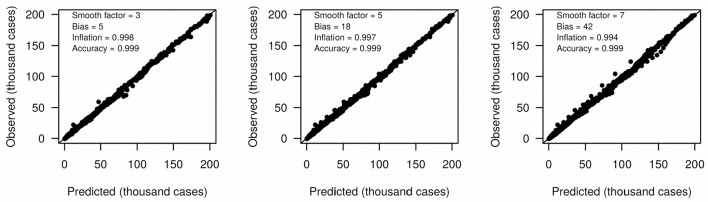
Accuracy (*R*^2^) of moving regression predictions of next-day COVID-19 prevalence.

We define growth rate and growth acceleration as the first and second order derivatives, respectively, of the prevalence of COVID-19 in respect to time. In our framework, we selected MR to approximate these derivatives over competing models that are frequently used to describe the behavior of sigmoidal growth curves, such as the Gompertz model (14, 15), because: (i) it is dependent on a single free parameter, the “smooth factor,” which represents the number of neighboring days used in local regression; (ii) growth rate and acceleration estimates are approximated by ordinary least squares equations, which are computationally inexpensive; (iii) we performed extensive simulations of growth curves and found that it produces reasonably accurate estimates of growth rate (median *R*^2^ = 0.99 with smooth factor of 3) and acceleration (median *R*^2^ = 0.92 with smooth factor of 3) (Figure 2); (iv) it is very robust to departures from sigmoidal curves; and (v) it does not rely on observations of the whole curve to produce instantaneous growth rate and acceleration estimates, and thus can produce such estimates in near real time. Argument (v) is especially relevant to the analysis of COVID-19 data since the pandemic is ongoing and each country will be at a different stage of the growth curve as time passes. A clear disadvantage of MR is that it may over-fit the growth curve to the data, especially if the selected smooth factor is small (say <3), in which case accurate prediction of new cases of COVID-19 is limited to very few days in the future. Still, even single-day predictions can be of great use during a pandemic if reasonably accurate. In the ECDC data set, a forward validation showed that single-day predictions were sufficiently accurate (*R*^2^ > 0.99) (Figure 3).

Sigmoidal growth curves can be partitioned into four easily distinguishable stages (Figure 1A): (a) the lagging stage, which corresponds to the beginning of the outbreak or disease importation, where the number of cases are low and increase only marginally every day; (b) the exponential stage, when growth starts accelerating and the number of new cases increase rapidly day-by-day; (c) the deceleration stage, where the number of new cases reduces daily and tends to asymptote; and (d) the stationary stage, characterized by stagnation of the prevalence with sporadic new cases occurring each day. The growth rate graph is approximately bell-shaped, with its peak corresponding to the inflection of the exponential stage. This inflection point signals the beginning of a decline in the growth rate. The growth acceleration graph usually consists of a combination of two bell-shaped curves: the first one with a peak and the second with a valley. The peak indicates the point where acceleration starts descending toward zero. The moment when acceleration is exactly zero coincides with the inflection of the exponential stage, which marks the beginning of growth deceleration (i.e., negative acceleration). The latter corresponds to the entire concave section of the curve, but the very bottom of the valley indicates that the prevalence is moving toward stagnation.

In spite of sigmoidal curves following the four above described stages sequentially, we anticipated that the growth of COVID-19 cases may not necessarily obey this sequence in practice, since the dynamics of the disease is likely complex and highly responsive to the implementation or relaxation of public health measures. This implies that a country that has already reached a stationary stage could resume exponential growth, for example by seeding a new outbreak via importation. Likewise, decelerating countries could as well regain acceleration by relaxing prevention measures. Furthermore, some countries may face multiple cycles of acceleration and deceleration prior to reaching a stationary growth. These scenarios could produce more complex growth curves that deviate from the sigmoidal shape by mounting different arrangements of exponential, deceleration, and stationary stages. Of note, MR has sufficient flexibility to model these complex scenarios and can easily accommodate curves exhibiting arbitrary permutations of these four stages. In addition, the near-zero acceleration that is intimately related to the stationary stage in sigmoidal curves could also arise from a non-zero constant growth rate in practice. In such cases, the growth curve would exhibit a linear pattern, which can be interpreted as a fifth growth stage that is not observed in classic sigmoidal functions. Such linear pattern may appear if the deceleration stage does not form an enough deep valley prior to acceleration rising up again toward zero. Again, MR is capable of modeling these anomalous behaviors. In this study we sought to ascertain whether these five stages of growth curves could have direct implications in understanding the dynamics of COVID-19 prevalence both globally and locally. We further developed a HMM to automate the detection of transitions between stages in the growth curve using acceleration and growth rate data obtained with MR as input (see **Material and Methods**).

**Figure 4:**
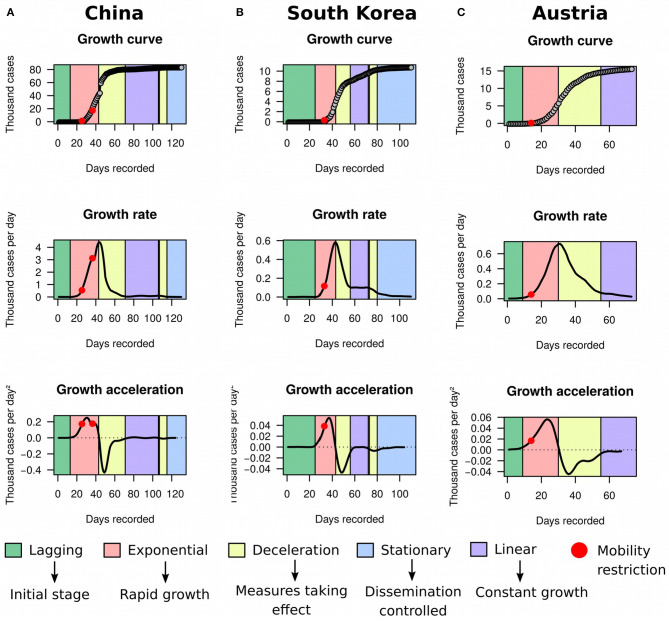
Growth rate and acceleration in China, South Korea, and Austria. **(A)** Fitted curves for China between December 31st 2019 and May 8th 2020. The first red dot marks the midpoint between January 23rd and 24th 2020, when a strict cordon sanitaire was imposed to Wuhan, Shanghai, Jiangsu, and Hainan. The second red dot pinpoints February 4th 2020, when the cordon was extended to a larger portion of the eastern part of China. **(B)** Fitted curves for South Korea between January 20th and May 8th 2020. The red dot is placed between February 20th and 21st, when a collection of restrictions to human mobility was imposed, including lockdown of Daegu city, suspension of flights, cancellation of mass gatherings, and lockdown of all South Korean military bases. **(C)** Fitted curves for Austria between February 26th and May 8th 2020. The red dot is placed on March 10th, when the Austrian government ordered children to stay at home and announced closure of universities and cancellation of public gatherings. The apparent stationary phase in these three countries was in reality classified as a mixture of linear growth, deceleration, and stationary stage by our framework.

Using MR and HMM on ECDC data frozen on May 8th 2020, we first evaluated the utility of the framework in identifying countries reaching stationary growth. Apart from Australia (Figure 1B) and New Zealand (Figure 1C), China (Figure 4A), South Korea (Figure 4B), and Austria (Figure 4C) also appeared to have reached stationary growth. However, our HMM classifier categorized the apparent stationary phase of these countries as a mixture of linear growth, deceleration, and stationary growth. Indeed, these three countries did not present a perfect asymptote after first deceleration, and their accumulated cases of COVID-19 were instead growing in a linear pattern for several days. China and South Korea further reached a stationary stage, but underwent an additional deceleration phase before. This implies that the growth dynamics of COVID-19 cases could be more complex than previously appreciated. Therefore, analyzing the raw growth curve alone, dissociated from its derivatives, is very limiting for inference and may hamper the understanding of the evolution of the pandemic.

By projecting government responses recorded by the Blavatnik School of Government from the University of Oxford (16) against the growth curves, we further observed that decline in growth acceleration occurred shortly after the implementation of measures that drastically reduced human movement. Upon restriction, decline in growth acceleration was typically detected within 1 week, deceleration of growth was achieved within 2 weeks, and the prevalence plateaued within 6 weeks. These results suggested that: (i) the effect of public health measures on SARS-CoV-2 prevention could be detected in seemingly real time by monitoring the behavior of acceleration curves; and (ii) restriction to human mobility is very effective in controlling the spread of the disease, but takes several weeks to produce a stationary growth. Indeed, regression of percent change in acceleration against policy indicators recorded in the Oxford dataset (Table 1) revealed that all indicators of mobility restriction were significantly associated with reductions in acceleration (*P* < 0.05). These findings are in line with a recent study showing that human mobility explained early growth and decline of new cases of COVID-19 in China (17).

**Table 1:** Effect of mobility restrictions on variation of COVID-19 acceleration (cases/day^2^) during exponential growth^a^.

**Policy indicator**	**Oxford code**	**Average change in acceleration after implementation (%)^b^**	**Standard error (%)**	***P*-value**
School closing	C1	−15.00	1.61	4.66 × 10^−20^
Workplace closing	C2	−14.25	1.25	4.33 × 10^−29^
Cancellation of public events	C3	−9.33	1.77	1.53 × 10^−7^
Restrictions on gatherings	C4	−10.62	1.30	7.68 × 10^−16^
Closure of public transportation	C5	−5.97	0.97	8.16 × 10^−10^
Stay at home requirements	C6	−7.67	1.11	7.18 × 10^−12^
Restrictions on internal movement	C7	−13.43	1.19	1.96 × 10^−28^
International travel controls	C8	−6.05	1.42	2.31 × 10^−5^

a *This analysis was performed with data from a subset of 62 countries presenting a minimum of 30 observed days and at least one exponential stage*.

b *Estimated from a linear regression of daily percent changes in acceleration against an indicator variable assuming value 1 if the policy is present and 0 otherwise*.

**Figure 5:**
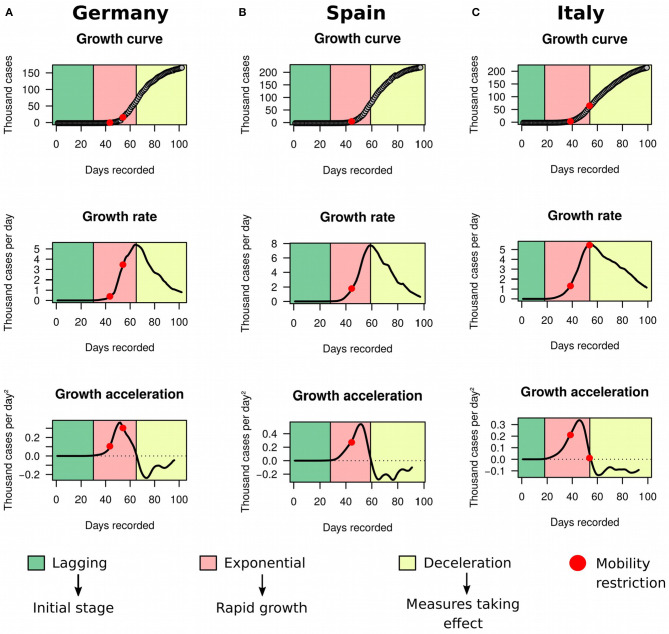
Growth rate and acceleration in Germany, Spain and Italy. These three countries were in deceleration as of May 8th. **(A)** Germany determined school closing in early March (first red dot) and extended restrictions to movement and gatherings within the country by March 22nd (second red dot). **(B)** Spain declared state of emergency on March 14th (red dot). Acceleration decline started 1 week later. **(C)** Italy imposed a strict quarantine on March 10th 2020 (first red dot) and closure of borders on March 25th 2020 (second red dot).

In order to illustrate the utility of the framework in detecting deceleration in real-time, we decided to look more closely to data from three countries: Germany (Figure 5A), Spain (Figure 5B), and Italy (Figure 5C). The latter has been severely impacted with the disease, and by the time we completed our study the country had recorded 215,858 cases and 29,958 deaths. On March 10th 2020, Italy implemented a strict quarantine. Five days later, the country reached its maximum acceleration and started to move toward an inflection of the exponential growth. On March 25th, Italy further implemented a complete shut down of its borders, and our analysis showed that the country started to decelerate on March 26th. In contrast, Germany applied a package of measures that started with school closing in early March and culminated in restrictions to internal movement and gatherings by March 22nd. Germany began deceleration on April 1st. Spain followed similar steps, with a state of emergency issued on March 14th. Acceleration decline started on March 22nd and deceleration began on March 31st in the country.

The relatively rapid response to public health measures makes the acceleration curve an useful tool for policy evaluation. Much attention has been recently given to Brazil and the United States of America (USA), as these two countries are the new epicenters of the coronavirus pandemic. Together, these two countries sum up 1,392,078 cases and 84,816 deaths to date. Monitoring the acceleration curve might be helpful in these countries by enabling the assessment of the efficacy of adopted measures. Since the beginning of the exponential growth in Brazil back in early March (data not shown), growth acceleration has presented great oscillation in the country. Currently, Brazil is experiencing an acceleration decline, and could begin a deceleration process within few weeks if effective measures are implemented and rigorously followed. On the other hand, USA has started its deceleration process on April 9th but has not formed a deceleration valley yet (data not shown), which hampers the production of an expressive decline in new cases. Furthermore, as outbreaks are expected to occur in African countries in the following months, the analysis of growth acceleration could be an invaluable asset to evaluate control strategies in the continent.

To this date, the lack of combined analysis of growth rate and acceleration of the COVID-19 pandemic is to be blamed on scarce availability of tailor made, user-friendly software. To aid to the analysis of growth rate and acceleration of COVID-19 cases, we built a web application using *R* (11) and *Shiny* (10). This application automatically loads the latest ECDC case reports and applies MR to extract growth rate and acceleration from real-time data. The app also performs automated classification of growth stages with HMM (albeit free parameters should be manually tuned for improved results). Users are not limited to case reports from ECDC, since the app allows for the upload of custom data (e.g., city, region, province, or state), which can be used to monitor the growth behavior of COVID-19 locally. Upon closing of the COVID-19 pandemic, this tool could be further used in the analysis of future outbreaks and epidemics, or even of historical disease data. A limiting factor however is that the proposed framework relies on updated case reports, such that sub-notification, delayed communication, and the elapsed time between sample collection, diagnostic results, and reporting may impact the real-time inference of growth dynamics in disease transmission and consequently jeopardize the timely detection of transitions in the growth curve. In spite of that limitation, the presented tool remains highly useful to monitor the growth behavior of epidemics.

## Conclusions

We deployed a simple framework for the real-time analysis of COVID-19 prevalence. We were able to demonstrate that the real-time decomposition of growth curves of COVID-19 cases into growth rate and acceleration can be a powerful tool to monitor the impact of public health measures on the spread of the disease. We also showed that restrictions to human mobility can significantly decelerate the incidence of new cases within weeks. Furthermore, we found that the prevalence of the disease is more complex and dynamic than previously appreciated. This observation will have important implications to assumptions adopted in mathematical models to predict the evolution of the pandemic.

## Materials and Methods

### Moving Regression (MR) Model

The MR technique (8) adopted here aimed at fitting a smooth growth curve to the COVID-19 prevalence data, such that the resulting curve could describe the cumulative number of cases as a function of time. For *n* recorded days in a given country or territory, let **x** be a *n*-dimensional column vector of days since the first case report and **y** the reciprocal column vector with elements corresponding to the cumulative number of cases. Relative to day *d*, we define **y**_d_ and **x**_d_ as *k*-sized subset vectors of **y** and **x**, respectively, where *k* = 1 + 2*s* and *s* is a free parameter representing the number of offset days before and after day *d*. Hereafter, we refer to *s* as the “smooth factor,” since it controls the compromise between over-smoothing (large *s*) and over-fitting (small *s*) the curve to the data. Finally, we define **X**_d_ = [**1**_k_ **x**_d_], where **1**_k_ is a *k*-dimensional column vector with all elements equal to one. The local growth rate was estimated by ordinary least squares regression:

(1) $$\begin{array}{l} {\left[\mu_{\text{d}}\;g_{\text{d}}\right]}^{\text{T}}={\left({\text{X}}_{\text{d}}^{\text{T}}\;{\text{X}}_{\text{d}}\right)}^{-1}{\text{X}}_{\text{d}}^{\text{T}}\;{\text{y}}_{\text{d}}\; \end{array}$$

where μ_d_ is an intercept and *g*_d_ is the estimated growth rate (cases/day) at day *d*. In practice, *g*_d_ corresponds to an estimate of the instantaneous rate of change in the number of cases at day *d*, which in turn is an approximation to the first order derivative of the unknown growth function evaluated at time *d*. The smoothed growth curve was obtained by calculating fitted values as:

(2) $$\begin{array}{l} {ŷ}_{\text{d}}={\text{X}}_{\text{d}}{\left[\mu_{\text{d}}\;g_{\text{d}}\right]}^{\text{T}}\; \end{array}$$

After fitting Equation (1) to all *n* records, we define **g** as a vector of size *n* containing all estimated local growth rates and **g**_d_ as a *k*-sized subset vector of **g**. The local growth acceleration at day *d* was then obtained by adapting Equation (1):

(3) $$\begin{array}{l} {\left[\mu_{\text{d}}a_{\text{d}}\right]}^{\text{T}}={\left({\text{X}}_{\text{d}}^{\text{T}}\;{\text{X}}_{\text{d}}\right)}^{-1}{\text{X}}_{\text{d}}^{\text{T}}\;{\text{g}}_{\text{d}}\; \end{array}$$

where *a*_d_ is the estimated growth acceleration (cases/day^2^) at day *d*. Now *a*_d_ is an estimate of the instantaneous rate of change of the growth rate at day *d*, which consequently approximates the second order derivative of the unknown growth function evaluated at time *d*.

### Hidden Markov Model (HMM) for Growth Stage Classification

In order to automate the process of growth stage classification, we built a HMM (9) that uses acceleration data obtained from MR as input. Considering **a** as the *n*-dimensional vector of estimated growth accelerations across *n* recorded days, we first compute **z** = sign(**a**), where sign(.) is a modified sign function which retrieves −1 for *a* < –*c*, +1 for *a* > *c* and 0 otherwise. Scalar *c* is defined as an acceleration cutoff, which is treated here as a free parameter. Through trial and error with both simulated and real data, we adopted a default value of *c* = 5 cases/day^2^. However, as a free parameter, *c* can be controlled by the user in order to obtain improved classification results. The objective of the HMM was to generate a sequence of states *K* = (*k*_1_, *k*_2_, …, *k*_n_) where each element *k*_i_ takes one of the following values: “lagging,” “exponential,” “deceleration,” or “stationary.” The initial probabilities for these hidden states were set to 1, 0, 0, and 0, respectively, assuming that all growth curves start from a lagging stage. Now let **T** be a 4 × 4 matrix of transition probabilities between hidden states and **E** be a 4 × 3 matrix of emission probabilities that models the probability of each hidden state producing a value of *z* of −1, 0, or +1. We adopted:

(4) $$\begin{array}{l} \text{T}=\left[\begin{array}{cccc} 0.8 & 0.2 & 0.0 & 0.0\\ 0.0 & 0.8 & 0.2 & 0.0\\ 0.0 & 0.0 & 0.8 & 0.2\\ 0.0 & 0.2 & 0.0 & 0.8 \end{array}\right]\text{E}=\left[\begin{array}{ccc} 0.25 & 0.40 & 0.25\\ 0.00 & 0.10 & 0.50\\ 0.50 & 0.10 & 0.00\\ 0.25 & 0.40 & 0.25 \end{array}\right]\; \end{array}$$

The selected values in **T** only permitted transitions lagging → exponential, exponential → deceleration, deceleration → stationary or stationary → exponential. Values in **E** made *z* = 0 more likely to be produced by either the lagging or stationary stages, *z* = +1 more likely to be produced by the acceleration stage and *z* = −1 more likely to be produced by the deceleration stage. For the atypical transition deceleration → exponential, the described model would generate a short and intermediate stationary step between these two stages. In these cases, the spurious stationary step was replaced by an exponential classification after the HMM has been fitted to the data. The Viterbi algorithm implemented in the HMM v1.0 package (18) in *R* (11) was used to estimate the sequence *K*. After prediction of growth stages, stationary classifications were confronted against growth rates. If a given stationary stage presented a median growth rate greater than the maximum growth rate of the lagging phase, it was re-classified as a “linear” stage. Again, values in matrices **T** and **E** were selected based on trial and error. We acknowledge that setting fixed values for **T** and **E** may limit the ability of the classifier in accommodating atypical transitions. Therefore, more flexible systems that calibrate these probabilities according to the observed data should be targeted in the near future.

### Simulation Study

To test the performance of MR in approximating growth curves and their rate of change and acceleration in scenarios where these curves have been observed only partially (i.e., real-time case report), we selected a widely used sigmoidal mathematical function, namely the Gompertz model (14, 15), to generate 50,000 simulated growth curves. We used a parameterization of the Gompertz model that is dependent on three parameters, apart from time:

(5) $$\begin{array}{l} f\left(t\right)=\alpha^{*}exp\left(-exp\left(-k\left(t-\delta \right)\right)\right) \end{array}$$

where *t* is a time point, α is the asymptote (i.e., number of cases at the stationary stage), exp is the exponential function, κ is a growth coefficient and δ is the time at inflection of the exponential stage (i.e., time when the growth rate reaches its maximum value and acceleration transitions from positive to negative). All simulations were performed considering a 100-days period, with parameters sampled as follows: α ~ Uniform(500, 10,000), κ ~ Uniform(0.05, 0.95), and δ ~ Uniform(5, 95). Completely stationary curves were discarded. The accuracy of growth rate and acceleration estimates produced by MR with smooth factor ranging from *s* = 3 to *s* = 10 were then evaluated by taking the coefficient of determination (*R*^2^) of the regression of true values onto estimates.

### Analysis of COVID-19 Case Reports

We analyzed case reports that have been updated daily by the European Center for Disease Prevention and Control (ECDC). The framework was applied to that data using smooth factors ranging from *s* = 3 to *s* = 10. The acceleration curves were clipped at observation *n* – *s* to avoid poor growth acceleration estimates at the end of the curve. Likewise, the last *s* days had their growth rates estimated by compounding rates from *n* – *s* to *n* using the acceleration estimated for day *n* – *s*. Finally, next-day predictions of COVID-19 prevalence were obtained by summing the last observed prevalence with its estimated growth rate. In order to measure the accuracy of these predictions, we performed a step-wise simulation by censoring observations ahead of each day, fitting MR to the remaining data and then comparing predicted and true next-day prevalence. Accuracy of predictions were again measured by linear regression.

### Analysis and Visualization Tools

All analyses presented in this paper were performed using *R* version 3.4.4 (11). To visualize the growth rate and acceleration of COVID-19 pandemic, we implemented a simple *Shiny* (10) dashboard application, which offers an intuitive web interface and allow us to be updated on new cases and the prevalence of COVID-19 worldwide. The application automatically loads the latest case reports from ECDC. Alternatively, users can upload their own data to visualize the growth rate and acceleration of COVID-19 of specific states, provinces, cities, or aggregate data from arbitrary territory definitions. For the implementation we used the following packages: shiny v1.4.0 (19), shinydashboard v0.7.1 (20), shinydashboardPlus v0.7.0 (21), readxl v1.3.1 (22), shinyalert v1.0 (23), httr v1.4.1 (24), and plotly v4.9.2 (25), all available on CRAN (Comprehensive R Archive Network, https://cran.r-project.org/). The application can be downloaded from our GitHub repository at https://github.com/adamtaiti/SARS-CoV-2/. A live instance of the app will be maintained until the end of the pandemic at https://theguarani.com.br/.

## Data Availability Statement

The COVID-19 case data in this study were obtained from the European Center for Disease Prevention and Control (ECDC) and are publicly available at https://opendata.ecdc.europa.eu/covid19/casedistribution/csv (accessed on May 8th 2020). Data on government responses were obtained from the Blavatnik School of Government, University of Oxford, and are available at https://www.bsg.ox.ac.uk/research/publications/variation-government-responses-covid-19 (accessed on May 8th 2020). The source code for the *R Shiny* application used for data analysis is found in our GitHub repository: https://github.com/adamtaiti/SARS-CoV-2. A live instance of the app can be accessed at https://theguarani.com.br/.

## Author Contributions

YU conceived the study, performed simulations, coordinated the data analysis, and wrote the manuscript. AU built R code for data analysis and programmed the Shiny App Dashboard. RT, SP, MM, and JG revised growth curves for all countries/territories and pinpointed dates of measures taken by them to reduce human mobility. All authors revised and agreed with the contents of the manuscript.

## Conflict of Interest

The authors declare that the research was conducted in the absence of any commercial or financial relationships that could be construed as a potential conflict of interest.
